# The Gain-of-Function R222S Variant in *Scn11a* Contributes to Visceral Hyperalgesia and Intestinal Dysmotility in *Scn*11*a*^R222S/R222S^ Mice

**DOI:** 10.3389/fneur.2022.856459

**Published:** 2022-05-27

**Authors:** Chenyu Zhao, Jishuo Jin, Haoye Hu, Xi Zhou, Xiaoliu Shi

**Affiliations:** ^1^Department of Gastroenterology, The Second Xiangya Hospital, Central South University, Changsha, China; ^2^Department of Medical Genetics, The Second Xiangya Hospital, Central South University, Changsha, China; ^3^Chigene (Beijing) Translational Medical Research Center Co., Ltd., Beijing, China; ^4^The National & Local Joint Engineering Laboratory of Animal Peptide Drug Development, College of Life Sciences, Hunan Normal University, Changsha, China

**Keywords:** *SCN11A*, Nav1.9 channel, intestinal dysmotility, visceral hyperalgesia, familial episodic limb pain

## Abstract

**Background:**

The *SCN11A* gene encodes the α-subunit of the Nav1. 9 channel, which is a regulator of primary sensory neuron excitability. Nav1.9 channels play a key role in somatalgia. Humans with the gain-of-function mutation R222S in *SCN11A* exhibit familial episodic pain. As already known, R222S knock-in mice carrying a mutation orthologous to the human R222S variant demonstrate somatic hyperalgesia. This study investigated whether *Scn*11*a*^R222S/R222S^ mice developed visceral hyperalgesia and intestinal dysmotility.

**Methods:**

We generated *Scn*11*a*^R222S/R222S^ mice using the CRISPR/Cas9 system. The somatic pain threshold in *Scn*11*a*^R222S/R222S^ mice was assessed by Hargreaves' test and formalin test. The excitability of dorsal root ganglia (DRG) neurons was assessed by whole-cell patch-clamp recording. Visceralgia was tested using the abdominal withdrawal reflex (AWR), acetic acid-induced writhing, and formalin-induced visceral nociception tests. Intestinal motility was detected by a mechanical recording of the intestinal segment and a carbon powder propelling test. The excitability of the enteric nervous system (ENS) could influence gut neurotransmitters. Gut neurotransmitters participate in regulating intestinal motility and secretory function. Therefore, vasoactive intestinal peptide (VIP) and substance P (SP) were measured in intestinal tissues.

**Results:**

The R222S mutation induced hyperexcitability of dorsal root ganglion neurons in *Scn*11*a*^R222S/R222S^ mice. *Scn*11*a*^R222S/R222S^ mice exhibited somatic hyperalgesia. In addition, *Scn*11*a*^R222S/R222S^ mice showed lower visceralgia thresholds and slowed intestinal movements when compared with wild-type controls. Moreover, *Scn*11*a*^R222S/R222S^ mice had lower SP and VIP concentrations in intestinal tissues.

**Conclusions:**

These results indicated that *Scn*11*a*^R222S/R222S^ mice showed visceral hyperalgesia and intestinal dysmotility.

## Introduction

The *SCN11A* gene encodes the α-subunit of voltage-gated sodium channel subtype 1.9 (Nav1.9). Nav1.9 channels are highly expressed in nociceptive neurons of the dorsal root ganglia (DRG) and Dogiel type II neurons of the enteric nervous system (ENS) (1, 2). Nav1.9 channels could regulate the resting potential of the membrane and amplify subthreshold stimuli (3). Nav1.9 channels are involved in generating action potentials in neurons and regulating neuronal excitability.

In the past, Nav1.9 channels were mainly considered to be involved in the formation of pain sensing (4). Gain-of-function pathogenic mutations in *SCN11A* cause familial episodic pain, painful peripheral neuropathy, and congenital insensitivity to pain. To date, no loss-of-function *SCN11A* mutations have been reported to be disease causing (5). The sense of pain includes somatic pain and visceralgia. Some patients with familial episodic pain also experience abdominal pain except for somatalgia (6, 7).

Recently, Nav1.9 channels have been found to participate in regulating colonic motility. Enteric motility is mainly regulated by the ENS but it can also be influenced by automatic neurons, gut hormones, and neurotransmitters. The ENS consists of sensory neurons, interneurons, and motor neurons, such as excitatory motor neurons and inhibitory motor neurons. Nav1.9 channels are expressed in sensory/Dogiel type II neurons (2). The frequency of colonic movement was significantly higher in *Scn*11*a*^−/−^ mice than in controls (8). *Scn*11*a*^+/L799P^ mice carrying the orthologous mutation with L811P (gain-of-function) in humans were affected by congenital insensitivity to pain. *Scn*11*a*^+/L799P^ mice showed a small shift toward less frequent intestinal peristaltic movements (no statistical significance) (9). Some pathogenic *SCN11A* mutations also cause gastrointestinal dysmotility symptoms in patients (6, 7, 10, 11).

Our group previously reported a familial episodic pain pedigree with a gain-of-function p.R222S *SCN11A* mutation (NM 014139) (12). Here, we bred knock-in mice with the R222S mutant in Nav1.9 (mNav1.9) channels. These mice showed increases in thermal pain behaviors and inflammatory pain responses, consistent with the results reported by Okuda et al. (13). Moreover, we observed that *Scn*11*a*^R222S/R222S^ mice showed lower visceralgia thresholds and slowed intestinal movements when compared with wild-type (WT) controls. These results support a role for Nav1.9 channels in regulating the excitability of the ENS that mediates visceral pain and intestinal motility.

## Materials and Methods

### Generation and Validation of the Knock-in Pain Model Mouse

#### Generation of Nav1.9 Knock-in Mouse

The R222S mutation is located on transmembrane segment S4 in domain I (DI) of the human Na_v_1.9 (hNav1.9) α subunit, which is the allelic ortholog of the amino acid site, R222S, in the mNav1.9 protein. The mutation was introduced into the mouse *Scn11a* locus using the CRISPR/Cas9 system at the Nanjing Biomedical Research Institute of Nanjing University (Nanjing, China). The single guide RNA (sgRNA) targeting the region around the mouse *Scn11a* R222 locus was designed using the Optimized CRISPR Design web tool (14). The sgRNA sequences are shown in Supplementary Table 1. Donor vectors carrying the *Scn11a* R222S mutation site fragment were generated. The donor vector and CRISPR/Cas9 system were microinjected into the fertilized ovum of C57BL/6 mice to generate *Scn*11*a*^+/R222S^ mice. Under the guidance of sgRNA, the CRISPR/Cas9 system cut the DNA strands at the targeting site. Fragments carrying the R222S mutation were recombined to the target site by homologous recombination. The genotypes of the offspring were confirmed by Sanger sequencing using the primers, which are presented in Supplementary Table 2. The PCR products were sequenced on an ABI 3730XL Genetic Analyzer (Thermo Fisher Scientific, Inc., Waltham, MA, USA).

The knock-in and WT C57BL/6 mice were fed and given water *ad libitum*. They were housed in an air-conditioned room with a 12 h light/dark cycle (light from 7:00 to 19:00) and controlled temperature (23 ± 2°C) and humidity (55 ± 10%). All tests were conducted between 14:00 and 18:00. All experiments involved 8 mice at 6–8 weeks old weighing 18–20 g unless otherwise noted. The animal protocol was approved by the ethics committee of the Laboratory Animal Center of the Second Xiangya Hospital of Central South University (Changsha, China). All experimental procedures were performed according to the relevant guidelines and regulations.

#### Somatic Pain Threshold Testing

For the Hargreaves' test, hind-paw thermal withdrawal latencies were tested using the Plantar Test Analgesia Meter (IITC Inc, Life Science). The mice were placed in transparent plastic testing chambers on glass plates for at least 30 min before testing. When the mice were resting but not sleeping, a movable radiant light heat source located under the glass floor was used to heat the plantar surface in the middle area of the hind paw. When the mice felt pain and withdrew the hind paw, the heat source was turned off and the reaction time counter was stopped. The paw withdrawal latency of each mouse was tested 3 times with an interval of 5 min and averaged to determine the heat threshold. To prevent tissue damage, the cutoff time was set as 20 s.

In the formalin test, the mice were placed in transparent plastic testing chambers and acclimated to the experimental environment for 15 min before the tests. Formalin solution (5%, 20 μl) was injected into the plantar surface of the hind paw. The total time of licking and flinching behaviors was recorded and binned at 5-min intervals for 45 min after injection. The total time of pain response in phase I (0–5 min) and phase II (10–45 min) was summarized.

#### Whole-Cell Patch-Clamp Recording of DRG Neurons

Small diameter DRG neurons were isolated from male WT, *Scn*11*a*^+/R222S^, and *Scn*11*a*^R222S/R222S^ mice (6–8 weeks old) as previously reported (15). Two mice of each group were euthanized by decapitation. Approximately 10–14 DRGs from the spinal cords were immediately dissected. L4-S1 ganglia harboring pelvic afferents from the colon were included. Then the ganglia were dissociated with collagenase XI (Sigma -Aldrich, Merck KGaA, Darmstadt, Germany) at 37°C for 25 min in an incubation medium containing Earle's balanced salt solution (Sigma-Aldrich, Merck KGaA, Darmstadt, Germany). Then, DRG cells were dispersed using fire-polished Pasteur pipettes and centrifuged. The cells were subsequently seeded onto poly-L-lysine-coated coverslips and maintained in Gibco Dulbecco's Modified Eagle Medium (DMEM) (Gibco) containing 10% heat-inactivated fetal bovine serum (FBS, Gibco) and 1% penicillin/streptomycin at 37°C in a humidified incubator with 5% CO_2_.

Whole-cell patch-clamp recordings were acquired using the EPC-10 USB patch-clamp platform (HEKA Elektronik, Ludwigshafen/Rhein, Germany) and Patchmaster software (HEKA Elektronik, Ludwigshafen/Rhein, Germany) at room temperature (20–25°C). Fire-polished borosilicate glass electrodes with resistances of 2.0–3.0 MΩ were fabricated from 1.5-mm glass capillaries using a puller (PC-10; Narishige, Tokyo, Japan). Data were filtered at 5 kHz and sampled at 20 kHz. The whole-cell recording configurations were achieved over 5 min.

In the current-clamp model, the pipette solution contained (in mM) 140 KCl, 0.5 ethylene glycol bis (2-aminoethyl ether)-N,N,N',N'-tetraacetic acid (EGTA), 5 4–1-piperazineethanesulfonic acid (HEPES), and 2 Mg-(adenosine triphosphate (ATP; pH 7.3 adjusted with KOH), and the bath solution contained (in mM) 140 NaCl, 3 KCl, 2 MgCl_2_, 2 CaCl_2_, and 10 HEPES (pH 7.3 adjusted with NaOH). All chemical reagents for the intracellular and extracellular solutions were purchased from Sigma-Aldrich (Merck KGaA, Darmstadt, Germany). Action potential frequency was calculated by action potential numbers during step current injections (500 ms) from 0 to 240 pA with 20 pA increments and rheobases were collected.

### Visceral Hyperalgesia Detection

#### AWR Test

The mice (12 weeks; 20–25 g) were deprived of food for 12 h. Visceral hyperalgesia in response to colorectal distention (CRD) was assessed using the AWR test. Mice were briefly anesthetized with ether. A balloon was inserted into the descending colon, and a catheter was fixed to the base of the tail. The mice were allowed to acclimate after waking for 1 h prior to CRD. The minimum threshold pressures resulting in strong contraction of the abdominal muscles that lifted the abdomen of the mice off the platform were recorded. The pressures resulting in the mice arching their bodies and lifting their pelvic structures were also recorded. The tests were repeated 3 times with an interval of 5 min. The pressure values for each mouse were averaged to determine the threshold. To prevent tissue damage, 100 mmHg was set as the cutoff pressure (16, 17).

#### Acetic Acid-Induced Writhing Test

The mice were deprived of food for 24 h and placed in transparent plastic testing chambers for 15 min to adapt the test environment. Then, each mouse was intraperitoneally injected with 0.8% acetic acid (0.1 ml/10 g). The number of writhing actions (contractions of the abdominal muscles, accompanied by stretching) was counted 20 min after injection.

#### Formalin-Induced Visceral Nociception Test

The mice were deprived of food for 24 h, then received a glycerine enema (0.1 ml) using an Fr6 catheter to prepare the bowel. After adapting for 1 h, 10% of formalin (10 μl) was instilled into the colon using a capillary rubber hose (1.5 mm external diameter), 2 cm from the anal sphincter. Then, the mice were inverted and their anus was blocked for 1 min using a finger. Vaseline was smeared onto the perianal region to avoid local nerve stimulation. Mice were then placed in transparent plastic testing chambers and abdomen licking behaviors were observed for 60 min.

### Intestinal Dysmotility Testing

#### Carbon Powder Propelling Test

The mice were deprived of food for 24 h before testing. A charcoal meal was prepared as described previously with minor modifications (18). In total, 5 g of activated carbon, 10 g of sodium carboxymethyl cellulose, 8 g of cane sugar, 16 g of milk powder, and 8 g of starch were mixed together. Then, the mixture was slowly added to 250 ml of distilled water and agitated for 1 min. Finally, the total volume of the mixture was ~300 ml. The test meal was stored at −20°C. Two hours before use, the test meal was removed from the refrigerator and allowed to reach room temperature. The carbon powder propelling test was performed similarly to a previously reported procedure (19). After fasting, the mice were gavaged with the test meal (0.5 ml/20 g). Twenty minutes later, they were euthanized by decapitation. The small intestine was resected carefully without artificially stretching the tissue. The distance charcoal traveled along the gastrointestinal tract (GI) tract was measured and quantified as a percentage of the distance traveled. The intestinal propulsion rate was calculated as follows: intestinal propulsion rate = charcoal meal transmission length/total small intestine length × 100%.

#### Mechanical Recording of the Intestinal Segment *in vitro*

The mice (10–12 weeks; 20–25 g) were deprived of food for 12 h. A part of the small intestine (2–4 cm from the pylorus) was dissected. The isolated intestine was gently flushed and placed in Tyrode's solution (Coolaber Technology Co., Ltd., Beijing, China) at 4°C. Then, the two ends were fixed on the tonotransducer and hooked on the bottom of a 20 ml measuring cylinder in the constant temperature smooth muscle test system (Techman Co., Ltd., Chengdu, China) using surgical sutures. The Tyrode's solution in the measuring cylinder was previously warmed up to 37 ± 0.1°C in a bath and equilibrated with pumped air. The initial tension of the muscle was set to 1,000 ± 100 mg. The tissue was allowed to equilibrate for 5 min. Then, the number of contractions and area under the contraction curve were continuously recorded for 3 min using a BL-420F biological signal acquisition and analysis system (Techman Co., Ltd., Chengdu, China).

#### The Neurotransmitters Measurement in Intestinal Tissue

The concentrations of vasoactive intestinal peptide (VIP), substance P (SP), and noradrenaline (NE) in the intestinal tissue of the mice were measured using ELISA kits (ZCIBIO Technology Co., Ltd., Shanghai, China) according to the directions of the manufacturer. The absorbance at 450 nm was measured on an EL × 800 microplate reader (BioTek Instruments, Inc., Vermont, USA).

### Statistical Analyses

Data were analyzed with GraphPad Prism 7.0 (GraphPad Software, San Diego, CA, USA). All data were presented as the mean ± standard error of the mean (SEM). Statistical tests of significance were conducted by one-way analysis of variance (ANOVA) followed by Dunnett's multiple comparison test or two-way ANOVA followed by Tukey's multiple comparison test. The criterion for statistical significance was *p* < 0.05.

## Results

### Increased Somatic Pain Sensitivity in *Scn*11*a*^R222S/R222S^ Mice

#### Scn11a^R222S/R222S^ Mice Showed Increased Heat Pain Sensitivity Under Basal Conditions

Both our team and Okuda et al. (13) have found that the R222S mutation led to familial episodic pain. We constructed this knock-in mouse using the CRISPR/Cas9 method to study the molecular mechanisms of the pain-causing mutation. The nucleotide change in mouse genomic DNA in *Scn11a* was validated by Sanger sequencing (Supplementary Figure 1).

**Figure 1:**
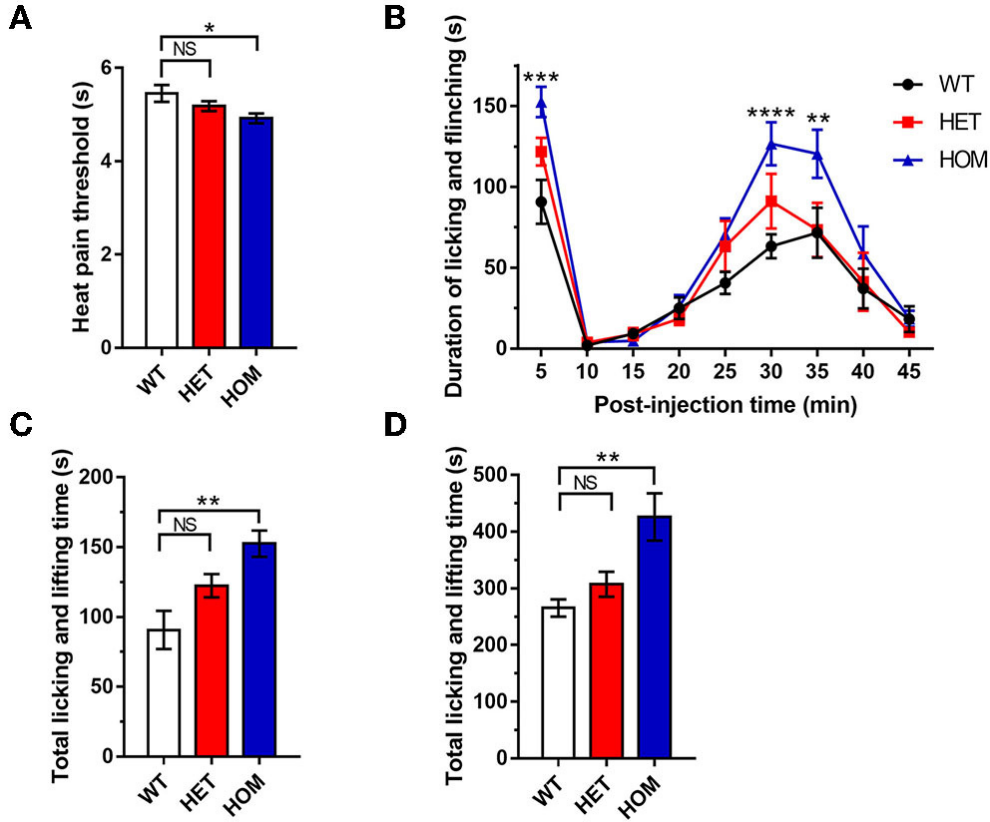
Somatic pain threshold comparison. **(A)** Hargreaves' test. Paw withdrawal latency was recorded using a radiant light heat source to heat the hind paw. **(B)** Formalin test. Total time spent licking and lifting the injected hind paw after intraplantar administration of formalin for 45 min, binned at 5-min intervals. **(C)** Data were summarized in Phase I (0–5 min) in the formalin test. **(D)** Data were summarized in phase II (10–45 min) in the formalin test. Data were presented as the mean ± SEM. Significance was tested with one-way ANOVA followed by the Dunnett's multiple comparisons test **(A,C,D)** or two-way ANOVA followed by the Tukey's multiple comparisons test **(B)**. *p < 0.05, **p < 0.01, ***p < 0.001, and ****p < 0.0001 vs. WT mice. NS, no significance; WT, wild-type (*Scn*11*a*^+/+^ mice); HET, heterozygote (*Scn*11*a*^+/R222S^ mice); HOM, homozygote (*Scn*11*a*^R222S/R222S^ mice).

We measured the heat pain threshold under basal conditions using Hargreaves' test. The heat withdrawal latency in *Scn*11*a*^R222S/R222S^ mice was significantly shorter than that in the WT group (WT mice: 5.5 ± 0.2 s, *n* = 8; *Scn*11*a*^+/R222S^ mice: 5.2 ± 0.1 s, *n* = 8; *Scn*11*a*^R222S/R222S^ mice: 4.9 ± 0.1 s, *n* = 8; *p* < 0.05, *Scn*11*a*^R222S/R222S^ vs. WT mice; Figure 1A). The *Scn*11*a*^R222S/R222S^ mice exhibited a lower heat pain threshold.

#### Formalin Aggravated Somatalgia in Scn11a^R222S/R222S^ Mice

To investigate pain threshold changes in inflammatory conditions, we performed a formalin test in 3 groups. A significant increase in paw licking and lifting time was observed in *Scn*11*a*^R222S/R222S^ mice during phases I and II when compared with WT mice (Figures 1B–D). However, the nociceptive behaviors in *Scn*11*a*^+/R222S^ mice were unaffected (phase I: WT mice: 90.8 ± 13.6 s, *n* = 8; *Scn*11*a*^+/R222S^ mice: 122.4 ± 8.3 s, *n* = 8; *Scn*11*a*^R222S/R222S^ mice: 152.5 ± 9.5 s, *n* = 8; *p* < 0.01, *Scn*11*a*^R222S/R222S^ vs. WT mice; Figure 1C; phase II: WT mice: 265.5 ± 15.3 s, *n* = 8; *Scn*11*a*^+/R222S^ mice: 307.1 ± 22.1 s, *n* = 8; *Scn*11*a*^R222S/R222S^ mice: 426 ± 41.8 s, *n* = 8; *p* < 0.01, *Scn*11*a*^R222S/R222S^ vs. WT mice; Figure 1D). Therefore, these results showed that the homozygotes were more sensitive to acute inflammatory pain.

#### The Dorsal Root Ganglion Neurons of Scn11a^R222S/R222S^ Mice Demonstrated Hyperexcitability

**Figure 2:**
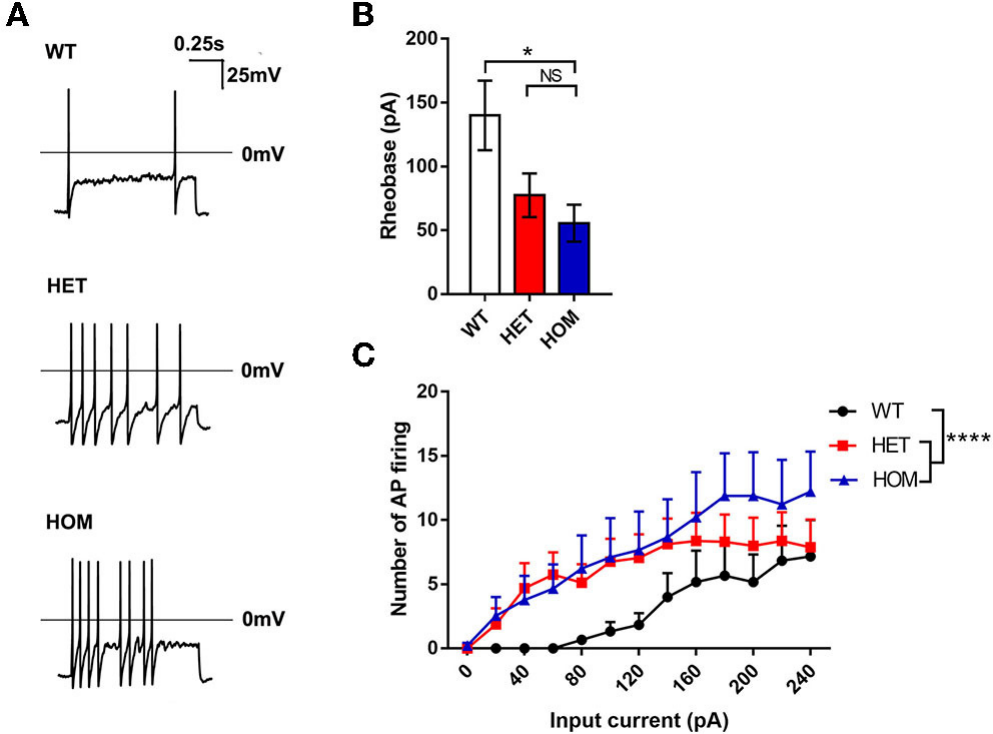
The R222S mutation increased the excitability of DRG neurons in electrophysiological examinations. **(A)** Action potential traces were recorded from a representative WT, HET, and HOM mouse DRG neuron, respectively. **(B)** The rheobases among 3 groups. **(C)** The number of action potential firing elicited by the depolarizing current steps from 0 to 240 pA in 20-pA increments in DRG neurons. Data were presented as the mean ± SEM. Significance was tested with one-way ANOVA followed by the Dunnett's multiple comparisons test **(B)** or two-way ANOVA followed by the Tukey's multiple comparisons test **(C)**. *p < 0.05 and ****p < 0.0001 vs. WT mice. NS, no significance; WT, wild-type (*Scn*11*a*^+/+^ mice); HET, heterozygote (*Scn*11*a*^+/R222S^ mice); HOM, homozygote (*Scn*11*a*^R222S/R222S^ mice).

The patch-clamp whole-cell recording technique was used to evaluate the excitability of DRG neurons. The examples of raw traces are shown in Figure 2A. The rheobase in *Scn*11*a*^R222S/R222S^ mice was significantly lower than that in WT mice, while the rheobase in *Scn*11*a*^+/R222S^ mice was similar to that in controls (WT mice: 140 ± 27.33 pA, *n* = 6; *Scn*11*a*^+/R222S^ mice: 77.5 ± 17.02 pA, *n* = 16; *Scn*11*a*^R222S/R222S^ mice: 55.56 ± 14.44 pA, *n* = 9; *p* < 0.05, *Scn*11*a*^R222S/R222S^ vs. WT mice; Figure 2B). The numbers of action potentials were increased with a series of current injections in *Scn*11*a*^R222S/R222S^ mice while firing frequency remained at a low level in WT mice (WT mice: *n* = 6, *Scn*11*a*^+/R222S^ mice: *n* = 16, and *Scn*11*a*^R222S/R222S^ mice: *n* = 9; *p* < 0.0001, *Scn*11*a*^+/R222S^ and *Scn*11*a*^R222S/R222S^ mice vs. WT mice; Figure 2C). The results indicated that DRG neurons from *Scn*11*A*^R222S/R222S^ mice evoked a higher frequency of action potential firing.

### *Scn*11*a*^R222S/R222S^ Mice Showed Visceral Hyperalgesia

#### The Visceral Mechanical Pain Threshold Decreased in Scn11a^R222S/R222S^ Mice

**Figure 3:**
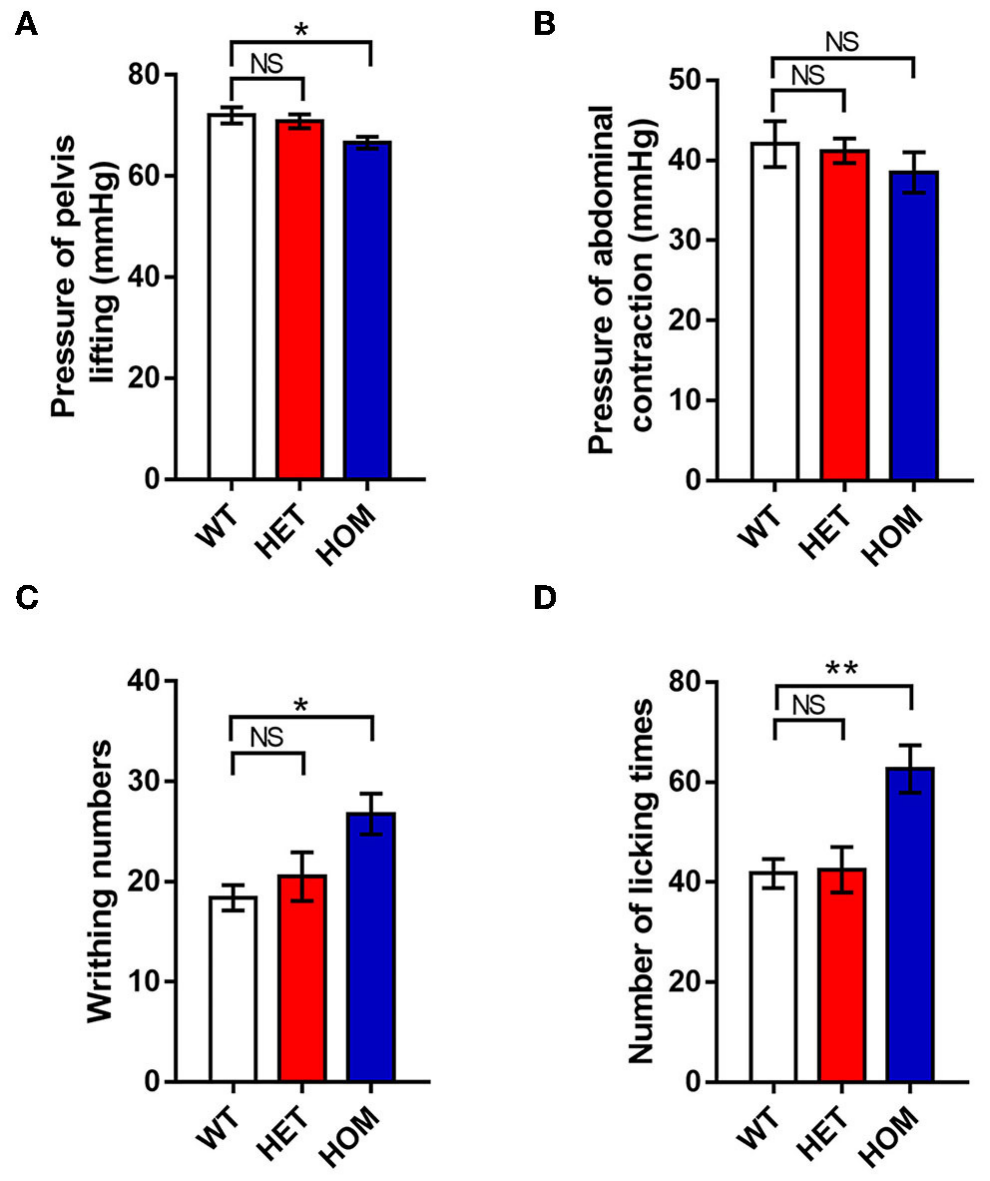
The visceral pain thresholds in different genotypes. **(A,B)** AWR test. The minimum thresholds of colorectal distention pressures were recorded when mice lifted the abdomen and pelvic structures. **(C)** Acetic acid-induced writhing test. The number of writhing actions (contractions of the abdominal muscles, accompanied by stretching) was counted in 20 min after intraperitoneal injection with acetic acid. **(D)** Formalin-induced visceral nociception test. The number of abdomen-licking behaviors was observed for 60 min after instilling formalin into the colon. Data were presented as the mean ± SEM. Significance was tested with one-way ANOVA followed by Dunnett's multiple comparisons test. *p < 0.05 and **p < 0.01 vs. WT mice. NS, no significance; WT, wild-type (*Scn*11*a*^+/+^ mice); HET, heterozygote (*Scn*11*a*^+/R222S^ mice); HOM, homozygote (*Scn*11*a*^R222S/R222S^ mice).

Most previous studies have focused on the relationship between Nav1.9 channels and peripheral somatosensory pain. However, in some cases, variants have also been found to cause visceral dysfunction. Next, we investigated whether there was also a correlation between the mutation and visceral dysfunction in the knock-in animal model. The AWR test was conducted to test the colonic mechanical pain threshold. The minimum threshold pressures of body arching and lifting the pelvic structure in the *Scn*11*a*^R222S/R222S^ group were significantly lower than those in WT groups (WT mice: 72.0 ± 1.6 mmHg, *n* = 8; *Scn*11*a*^+/R222S^ mice: 70.9 ± 1.4 mmHg, *n* = 8; *Scn*11*a*^R222S/R222S^ mice: 66.6 ± 1.2 mmHg, *n* = 8; *p* < 0.05, *Scn*11*a*^R222S/R222S^ vs. WT mice; Figure 3A). However, there were no significant differences in the minimum threshold pressure required to induce lifting the abdomen off the platform among the 3 groups (WT mice: 42.0 ± 2.9 mmHg, *n* = 8; *Scn*11*a*^+/R222S^ mice: 41.2 ± 1.5 mmHg, *n* = 8; *Scn*11*a*^R222S/R222S^ mice: 38.5 ± 2.5 mmHg, *n* = 8; *p* = 0.5, *Scn*11*a*^R222S/R222S^ vs. WT mice; Figure 3B). Taken together, these results suggested that *Scn*11*a*^R222S/R222S^ mice were more sensitive to colonic mechanical stimuli.

#### Acetic Acid Evoked More Serious Visceralgia in Scn11a^R222S/R222S^ Mice

Next, we investigated visceral sensitivity to acute inflammation using writhing experiments. Intraperitoneal application of acetic acid provoked a significant increase in writhing responses in homozygotes when compared with WT controls (WT mice: 18.4 ± 1.3, *n* = 8; *Scn*11*a*^+/R222S^ mice: 20.5 ± 2.4, *n* = 8; *Scn11a* ^R222S/R222S^ mice: 26.8 ± 2.0, *n* = 8; *p* < 0.05, *Scn*11*a*^R222S/R222S^ vs. WT mice; Figure 3C). Therefore, acute inflammation was more likely to cause visceral pain in homozygotes.

#### Formalin Instillation Into the Colon Aggravated Visceralgia in Scn11a^R222S/R222S^ Mice

To test visceralgia sensitivity to acute inflammation, nociceptive pain behaviors induced by formalin instillation into the colon were observed. Nociceptive pain behaviors were significantly more frequent in *Scn*11*a*^R222S/R222S^ mice than in normal controls (WT mice: 41.8 ± 2.9, *n* = 8; *Scn*11*a*^+/R222S^ mice: 42.5 ± 4.5, *n* = 8; *Scn11a* ^R222S/R222S^ mice: 62.6 ± 4.8, *n* = 8; *p* < 0.01, *Scn*11*a*^R222S/R222S^ vs. WT mice; Figure 3D). The data demonstrated that *Scn*11*a*^R222S/R222S^ mice had visceral hyperalgesia in response to acute inflammation.

### *Scn*11*a*^R222S/R222S^ Mice Manifested Intestinal Dysmotility

#### Scn11a^R222S/R222S^ Mice Presented With Longer Intestinal Transit Time *in vivo*

**Figure 4:**
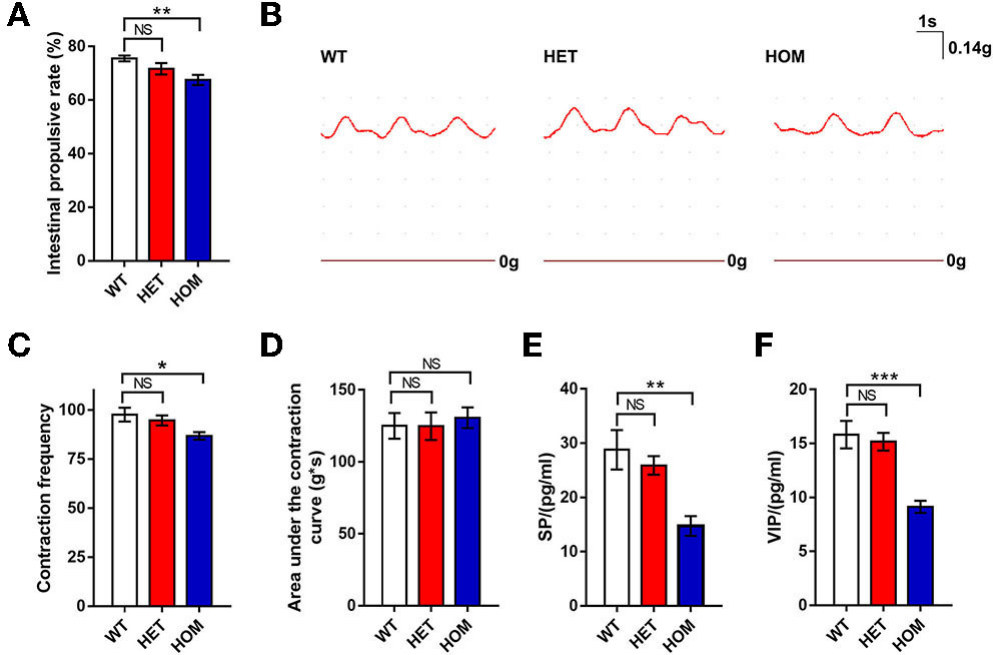
Intestinal motility comparison among 3 groups. **(A)** Carbon powder propelling test. Twenty minutes after gavage with a charcoal meal, distances traveled by charcoal meal along the intestine in mice were measured and quantified as a percentage of distance traveled. **(B)** The traces of the intestinal segments contraction curves were recorded from a representative WT, HET, and HOM mouse, respectively. **(C,D)** The tonotransducer continuously recorded the number of contractions and calculated area under the contraction curve for 3 min. **(E,F)** The concentrations of SP and VIP (tested by ELISA) were decreased in the intestinal tissues of *Scn11a* knock-in mice. Data were presented as the mean ± SEM. Significance was tested with one-way ANOVA followed by Dunnett's multiple comparisons test. *p < 0.05, **p <0.01, ***p < 0.001 vs. WT mice. NS, no significance; WT, wild-type (*Scn*11*a*^+/+^ mice); HET, heterozygote (*Scn*11*a*^+/R222S^ mice); HOM, homozygote (*Scn*11*a*^R222S/R222S^ mice).

The carbon powder propelling test was used to investigate the difference in intestinal motility among the 3 groups *in vivo*. The distances of the carbon powder traveled in the *Scn*11*a*^R222S/R222S^ group were significantly shorter than those in the control WT group (WT mice: 75.5 ± 1.1%, *n* = 8; *Scn*11*a*^+/R222S^ mice: 71.7 ± 2.2%, *n* = 8; *Scn11a* ^R222S/R222S^ mice: 67.5 ± 1.9%, *n* = 8; *p* < 0.01, *Scn*11*a*^R222S/R222S^ vs. WT mice; Figure 4A). The results indicated that intestinal tract movement was slower in *Scn*11*a*^R222S/R222S^ mice.

#### Intestinal Segments of Scn11a^R222S/R222S^ Mice Showed Less Peristalsis *in vitro*

The mechanical activity of small intestine segments was evaluated to investigate intestinal motility. The examples of raw traces are shown in Figure 4B. The number of contractions in intestinal segments from *Scn*11*a*^R222S/R222S^ mice was lower than that in those from WT mice, whereas the number of contractions in segments from *Scn*11*a*^+/R222S^ mice was not different (WT mice: 97.6 ± 3.5, *n* = 8; *Scn*11*a*^+/R222S^mice: 94.6 ± 2.6, *n* = 8; *Scn*11*a*^R222S/R222S^ mice: 86.8 ± 2.0, *n* = 8; *p* < 0.05, *Scn*11*a*^R222S/R222S^ vs. WT mice; Figure 4C). No significant difference in the area under the contraction curve was observed among the 3 groups (WT mice: 125 ± 8.9 g × s, *n* = 8; *Scn*11*a*^+/R222S^ mice: 124.8 ± 9.6 g × s, *n* = 8; *Scn*11*a*^R222S/R222S^ mice: 130.6 ± 7.2 g × s, *n* = 8; *p* = 0.9, *Scn*11*a*^R222S/R222S^ vs. WT mice; Figure 4D). These data revealed less frequent intestinal peristaltic movements in *Scn*11*a*^R222S/R222S^ mice.

#### VIP and SP Were Decreased in Intestinal Tissue From Scn11a^R222S/R222S^ Mice

To investigate the changes of neurotransmitters in the ENS, we measured SP, VIP, and NE by ELISA. The concentrations of SP in intestinal tissues in *Scn*11*a*^R222S/R222S^ mice were significantly lower than those in WT mice, while those in *Scn*11*a*^+/R222S^ mice were similar to those in controls (WT mice: 28.8 ± 3.6 pg/ml, *n* = 4; *Scn*11*a*^+/R222S^ mice: 25.9 ± 1.7 pg/ml, *n* = 7; *Scn*11*a*^R222S/R222S^ mice: 14.7 ± 1.8 pg/ml, *n* = 4; *p* < 0.01, *Scn*11*a*^R222S/R222S^ vs. WT mice; Figure 4E). *Scn*11*a*^R222S/R222S^ also showed significantly lower VIP concentrations than those in WT controls (WT mice: *n* = 5, 15.8 ± 1.3 pg/ml; *Scn*11*a*^+/R222S^ mice: *n* = 7, 15.2 ± 0.8 pg/ml; *Scn*11*a*^R222S/R222S^ mice: *n* = 5, 9.1 ± 0.6 pg/ml; *p* < 0.001, *Scn*11*a*^R222S/R222S^ vs. WT mice; Figure 4F). *Scn*11*a*^R222S/R222S^ mice showed a slight increment in NE concentrations in intestinal tissues. However, there were no significant differences among the 3 genotypes (Supplementary Figure 2).

## Discussion

The mechanical recording of the intestinal segment and carbon powder propelling test revealed enteral dysmotility. *Scn*11*a*^R222S/R222S^ mice showed a lower contraction frequency and longer small intestinal transit time than those in WT mice. In another study, a patient with the L811P gain-of-function mutation in *SCN11A* showed reduced small intestine peristaltic waves by laparotomy (11). *Scn11a*^+/*L*799*P*^ mice carrying mutation orthologous with the human L811P mutation show a small shift toward less frequent intestinal peristaltic movements (no statistical significance), and the gastrointestinal transit time was unaffected overall. However, *Scn11a* ^L799P/L799P^ mice were not tested (9). L811P and R222S are both gain-of-function mutations. However, the former causes pain insensitivity while the latter causes familial episodic pain. Despite these differences, both mutations are associated with the same tendency toward reduced intestinal peristaltic movements. In addition, some patients with gain-of-function *SCN11A* mutations experience constipation, diarrhea, or mixed symptoms, demonstrating opposite symptoms. Its mechanism was not clarified. Therefore, the functional impact of Nav1.9 mutations on ENS needs further investigation. *Scn*11*a*^+/L799P^ mice exhibited insensitivity to pain, but sensitivity to pruritus (11, 20). The functional influence of Nav1.9 in different sensory modalities also needs further study.

The SP secreted by excitatory motor neurons is an excitatory neurotransmitter in the ENS that causes intestinal smooth muscle contraction. Conversely, VIP is an inhibitory neurotransmitter that causes intestinal smooth muscle relaxation. VIP can also increase intestinal secretion. VIP tumors (Verner-Morrison syndrome) abnormally secret excess VIP. In patients, this increase in VIP causes secretory diarrhea (21). A rat model with constipation has been shown to have decreased expression of VIP levels in colon tissues (22). Overall, VIP can decrease transit time. In our study, *Scn*11*a*^R222S/R222S^ mice showed lower concentrations of SP and VIP. This result indicated a slower intestinal transmission speed in *Scn*11*a*^R222S/R222S^ mice.

Gastrointestinal sensory mechanisms play a crucial role in triggering motor reflexes by transmitting sensory information to the enteric reflex circuits that perform local control *via* afferent pathways to the central nervous system (23). There are mainly four types of afferent neurons in the gut, i.e., primary afferent neurons with cell bodies in DRG, primary afferent neurons with cell bodies in vagal sensory ganglia, intrinsic primary afferent neurons (IPANs) in the ENS, and intestinofugal afferent neurons (IFANs) in the ENS (24). IFANs with Dogiel type II morphology project to sympathetic prevertebral ganglia (PVG) neurons, and their cell bodies are within enteric ganglia (25). In addition, IPANs mainly belong to Dogiel type II neurons (26). The Nav1.9 channel expresses in the ENS, which is located on Dogiel type II neurons in mice (2, 27), and may involve in regulating the neuronal excitability.

**Figure 5:**
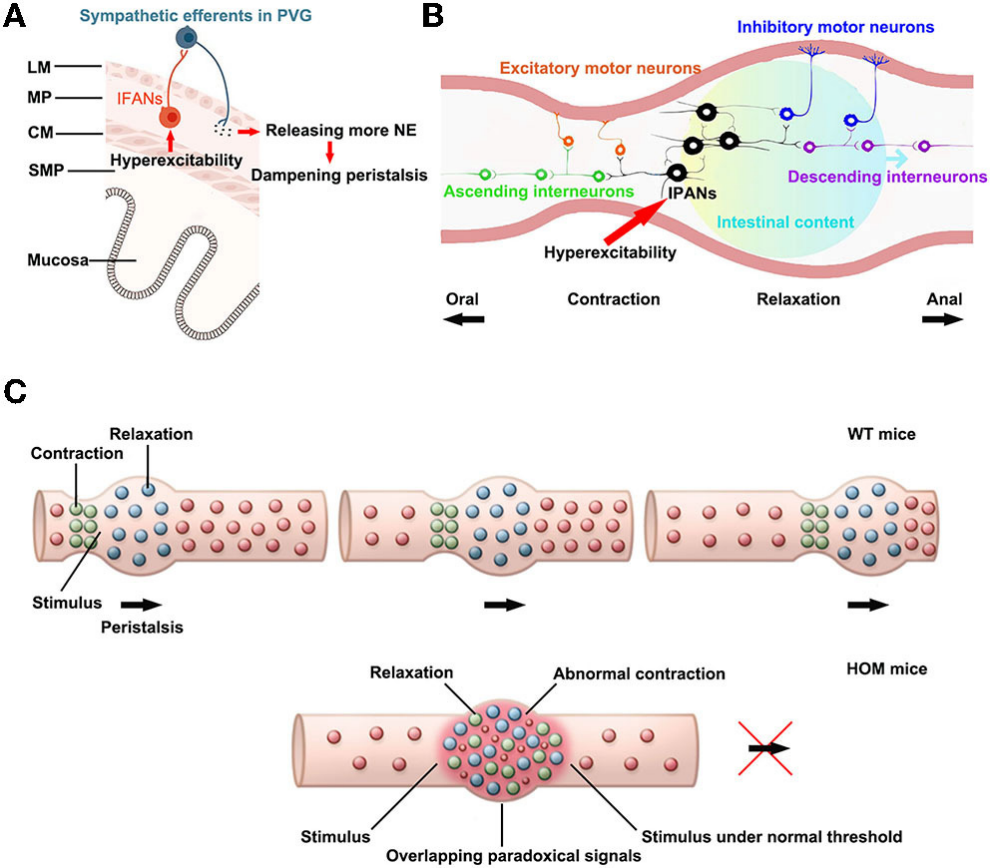
The potential mechanisms for hyperexcitability in sensory afferents lead to reduced intestinal motility. **(A)** The influence on entero-enteric inhibitory reflexes. PVG: prevertebral ganglion, IFANs: intestinofugal afferent neurons, NE: noradrenaline, LM: longitudinal smooth muscle, MP: myenteric plexus, CM: circular smooth muscle, SMP: submucosal plexus. The paragraph was modified from Spencer and Hu (26). **(B)** The alteration in peristalsis reflexes. IPANs: intrinsic primary afferent neurons. This paragraph was modified from Fung and Vanden Berghe (32). **(C)** The abnormal peristalsis reflexes triggered by hyperexcitability sensory neurons might disrupt intestinal peristalsis waves. WT, wild-type (*Scn*11*a*^+/+^ mice); HOM, homozygote (*Scn*11*a*^R222S/R222S^ mice). This paragraph was modified from Mawe (31).

Intestinofugal afferent neurons convey signals to sympathetic PVG neurons, and the activation of sympathetic PVG neurons could inhibit intestinal peristalsis (entero-enteric inhibitory reflexes) (24). The excess stimulation by visceral afferent (sensory) fibers could modulate motor neurons in PVG, which may influence local gastrointestinal motor function and induce dysmotility (28). The sympathetic neurons mainly release the neurotransmitter NE. Moreover, the NE could dampen peristalsis (29). Therefore, the hyperexcitability of IFANs might activate sympathetic neurons *via* PVG (Figure 5A). Nav1.9 channels in *Scn*11*a*^R222S/R222S^ mice had hyperexcitability. In addition, *Scn*11*a*^R222S/R222S^ mice showed a slight increment in NE concentrations in intestinal tissues. These data indicated that entero-enteric inhibitory reflexes in *Scn*11*a*^R222S/R222S^ mice might be abnormally activated.

The trinitrobenzene sulfonic acid (TNBS)-colitis induced hyperexcitability of myenteric afferent neurons. The TNBS-colitis model leads to temporarily halted motility or obstructed at sites of ulceration. Moreover, suppression of the excitability of afterhyperpolarization (AH) neurons/IPANs could restore colonic motility in guinea pigs *ex vivo* following the inflammation. These results support that enhanced excitability of AH neurons/IPANs could contribute to dampened propulsive colonic motility (30). The gain-of-function alteration of Nav1.9 in *Scn*11*a*^R222S/R222S^ mice also induced hyperexcitability of IPANs, which may reduce intestinal contractions.

In the intestinal peristalsis reflex, the mechanical and/or chemical stimulation causes activation of IPANs at the location of the stimulus. These IPANs, along with interneurons, convey signals ascendingly to activate excitatory motor neurons and descendingly to activate inhibitory motor neurons. The outcome is a pressure gradient, which propels the intestinal luminal contents distally (Figure 5B). Moreover, as the process repeats itself, the peristalsis wave is generated (31, 32).

In the regions of inflammation, IPANs are spontaneously active and synaptic activity is augmented. The alterations cause overlapping descending inhibitory and ascending excitatory signals in the regions. In addition, inhibitory neuromuscular transmission is decreased. Peristalsis is disrupted by the mixed signals and the suppressed neuromuscular transmission in the inflammation region (31). *Scn*11*a*^R222S/R222S^ mice also existed overactive firing in IPANs, which might lead to overlapping paradoxical signals. The mixed signals may contribute to disrupt normal pressure gradients in peristalsis (Figure 5C). However, due to the complexity of gastrointestinal regulations, the exact mechanisms linking the hyperexcitability of the sensory neurons with delayed peristalsis need further investigations.

*Scn*11*a*^R222S/R222S^ mice exhibited an enhanced response to heat and formalin stimulus. These results suggested a decreased somatic pain threshold in these mice. Additionally, *Scn*11*a*^R222S/R222S^ mice showed visceral hyperalgesia in the AWR test, acetic acid-induced writhing test, and formalin-induced visceral nociception test. The excitability of DRG neurons in *Scn*11*a*^R222S/R222S^ mice was higher than that in WT mice. DRG neurons are the primary neurons that conduct pain. Therefore, their hyperexcitability could cause somatic and visceral hyperalgesia. Another study also recognized that Nav1.9 channels play a key role in visceral pain by affecting nociceptive neurons (33).

## Conclusions

In conclusion, this study revealed that *Scn*11*a*^R222S/R222S^ mice have slower small intestine peristalsis than that in WT controls and increased visceral hypersensitivity. Our results indicate that the *Scn11a* gene contributes to the regulation of visceral sensitivity and intestinal motility.

## Data Availability Statement

The raw data supporting the conclusions of this article will be made available by the authors, without undue reservation.

## Ethics Statement

The animal study was reviewed and approved by Animal Ethical and Welfare Committee, the Second Xiangya Hospital, CSU, P.R. China.

## Author Contributions

CZ: formal analysis, investigation, methodology, software, and writing—original draft. JJ and HH: investigation. XZ and XS: conceptualization, supervision, validation, visualization, and writing—review and editing. All authors contributed to the article and approved the submitted version.

## Funding

This research was financially supported by the National Natural Science Foundation of China (Grant Nos. 31800655 and 81741051) and the Science and Technology Innovation Program of Hunan Province (Grant Nos. 2021RC3092 and 2016JC2045).

## Conflict of Interest

JJ is now employed by Chigene (Beijing) Translational Medical Research Center Co., Ltd., Beijing, China, however, at the time of participation in the investigation he was studying for a Master's Degree at the Second Xiangya Hospital. Chigene (Beijing) Translational Medical Research Center Co., Ltd., were not involved in the study design, collection, analysis, interpretation of data, the writing of this article or the decision to submit it for publication. The remaining authors declare that the research was conducted in the absence of any commercial or financial relationships that could be construed as a potential conflict of interest.

## Publisher's Note

All claims expressed in this article are solely those of the authors and do not necessarily represent those of their affiliated organizations, or those of the publisher, the editors and the reviewers. Any product that may be evaluated in this article, or claim that may be made by its manufacturer, is not guaranteed or endorsed by the publisher.
