# Supplementary material for: The Gain-of-Function R222S Variant in Scn11a Contributes to Visceral Hyperalgesia and Intestinal Dysmotility in Scn11aR222S/R222S Mice
Source: Front Neurol. 2022 May 27;13:856459. doi: 10.3389/fneur.2022.856459 (PMC9197071; doi:10.3389/fneur.2022.856459)
Supplement: Supplementary file 1 [file Presentation_1.pdf]

Supplemental Materials

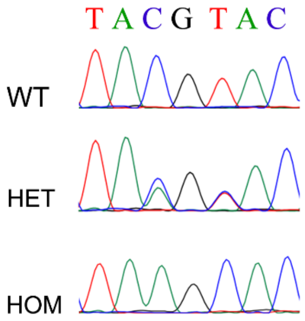

Figure S1. Genotypes verification of the knock-in mice

Validation of the genotypes from genomic DNA derived from tail samples by Sanger sequencing. WT: wild type (*Scn11a*$^{+/+}$ mice), HET: heterozygote (*Scn11a*$^{R222S/+}$ mice), HOM: homozygote (*Scn11a*$^{R222S/R222S}$ mice).

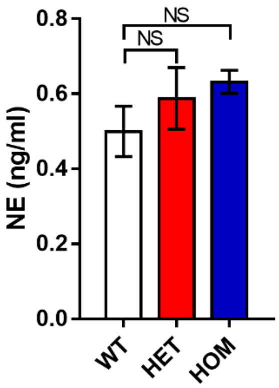

Figure S2. Concentrations of noradrenalin in intestinal tissues

There were no significant differences in the concentrations of noradrenalin in intestinal tissues (tested by ELISA) among 3 genotypes. *Scn11a*$^{\mathrm{R222S/R222S}}$ mice showed slight increasement in noradrenalin concentrations (WT mice: n = 5, 0.5 ± 0.07 ng/mL; *Scn11a*$^{\mathrm{+/R222S}}$ mice: n = 5, 0.63 ± 0.03 ng/mL; *Scn11a*$^{\mathrm{R222S/R222S}}$ mice: n = 5, 0.59 ± 0.08

ng/mL; P>0.05, *Scn11a*$^{R222S/R222S}$ vs. WT mice). Data were presented as the mean ± SEM. Significance was tested with one-way ANOVA followed by Dunnett's multiple comparisons test. NE: noradrenalin, NS: no significance, WT: wild-type (*Scn11a*$^{+/+}$ mice), HET: heterozygote (*Scn11a*$^{+/R222S}$ mice), HOM: homozygote (*Scn11a*$^{R222S/R222S}$ mice).

Table S1. SgRNA sequence

Sequence (5'-3')	Protospacer adjacent motif
CAGAGCTCTCAACACTCGGA AGG	AGG

Table S2. Primer sequences for mutation analysis of *Scn11a*

Primer	Sequence (5'-3')
Scn11a-mutant-F	GGCGCTATTCATCTCAGAGTT
Scn11a-mutant-R	CCCAGCTCAGAAATGTTCTAATGC
